# Editorial Comment to Identification of tumor immunophenotypes associated with immunotherapy response in bladder cancer

**DOI:** 10.1111/iju.15296

**Published:** 2023-09-04

**Authors:** Tibor Szarvas

**Affiliations:** ^1^ Department of Urology, Faculty of Medicine University Duisburg‐Essen Essen Germany; ^2^ Department of Urology Semmelweis University Budapest Hungary

Immune checkpoint inhibition (ICI) has emerged as a promising therapeutic approach for advanced tumors. In 2016 and 2017, atezolizumab and pembrolizumab, became available for patients with urothelial carcinoma (UC) that had progressed during or following platinum‐containing chemotherapy, providing significantly higher objective response rates (15%–29%), compared to other second‐line chemotherapies.^1^ Importantly, responding patients experienced a durable effect of over 12 months, which is a remarkable improvement at this stage. In 2018, both treatments became available in the first‐line setting for cisplatin‐ineligible patients with high programmed death‐ligand 1 (PD‐L1) immunostaining, while for those patients with low PD‐L1 expression carboplatin therapy is preferred.^2^ Currently, a further programmed cell death protein 1 (PD‐1) antibody, avelumab has been approved as a maintenance therapy for locally progressed or metastatic UC who responded or had disease stabilization after first‐line platinum‐containing chemotherapy.^3^ More recently, an antibody‐drug conjugate enfortumab‐vedotin received approval in the third‐line setting following platinum and ICI therapy.^4^ In addition, various ICIs and antibody‐drug conjugates are being tested alone or in combination in earlier settings. Overall, the treatment landscape for invasive UC is rapidly evolving, leading to increased complexity in therapeutic decision‐making. Therefore, predicting the efficacy of ICI is increasingly important.

Resistance to ICI has been shown to stem from tumor‐intrinsic elements, including genomic alterations, oncogenic signaling, and hindered antigen presentation. Additionally, tumor‐extrinsic factors like host immunity and the tumor microenvironment contribute to this resistance. While biomarkers and surrogates such as tumor mutational burden and PD‐L1 expression on tumor‐infiltrating immune cells have shown predictive value for ICI response in UC, they remain imperfect and lack the necessary robustness for routine clinical application.

Against this background, the recent study by Cheng et al. addressed the clinically important unmet need for the prediction of ICI efficacy.^5^ For this, authors assessed publicly available UC gene expression datasets to selected immune infiltration‐related genes. Using these genes they defined two distinct gene expression‐based immunophenotypes: *inflamed* and *excluded*, which were subsequently subjected to detailed analysis. This newly identified subtypes proved to be prognostic in the examined cohort, which could be confirmed in the independent dataset of the TCGA study. Notably, the *inflamed* subtype exhibited ICI predictive value by showing a significant correlation with more favorable radiographic response among UC patients who underwent ICI therapy in the IMvigor210 study cohort. This study contributes to the growing body of gene expression‐based studies. The potential predictive value of the *inflamed* subtype, pending confirmation through additional research, could potentially support more individualized clinical decision‐making informed by molecular insights in the changing therapeutic landscape of UC.

## CONFLICT OF INTEREST STATEMENT

The author declares no conflict of interest.
